# Comparative transcriptomes reveal molecular mechanisms of apple blossoms of different tolerance genotypes to chilling injury

**DOI:** 10.1515/biol-2022-0613

**Published:** 2023-12-28

**Authors:** Xiaolong Li, Haiying Yue, Yannan Chu, Yonghua Jia

**Affiliations:** Department of Plant Science, Institute of Horticulture, Ningxia Academy of Agricultural and Forestry Sciences, Yinchuan, 750000, Ningxia, China

**Keywords:** apple, chilling gene, cold stress, transcriptome, anti-oxidation

## Abstract

Apple (*Malus domestica*, Borkh.) is one of the four largest fruits in the world. Freezing damage during the flowering period of apples is one of the main factors leading to the reduction or even extinction of apple production. Molecular breeding of hardy apples is a good solution to these problems. However, the current screening of cold tolerance genes still needs to be resolved. Therefore, in this article, the transcriptome detection and cold tolerance gene screening during the cold adaptation process of apple were studied in order to obtain potential cold-resistant genes. Herein, two high-quality apple tree species (*Malus robusta* Rehd and *M. domestica*) were used for cold adaptation experiments and studied under different low-temperature stress conditions (0, −2 and −4°C). The antioxidant levels of two apple flower tissues were tested, and the transcriptome of the flowers after cold culture was tested by next-generation sequencing technology. Antioxidant test results show that the elimination of peroxides in *M. robusta* Rehd and the adjustment of the expression of antioxidant enzymes promote the cold resistance of this variety of apples. Functional enrichment found that the expression of enzyme activity, cell wall and cell membrane structure, glucose metabolism/gluconeogenesis, and signal transmission are the main biological processes that affect the differences in the cold resistance characteristics of the two apples. In addition, three potential cold-resistant genes AtERF4, RuBisCO activase 1, and an unknown gene (ID: MD09G1075000) were screened. In this study, three potential cold-resistant genes (AtERF4, RuBisCO activase 1, and an unknown gene [ID: MD09G1075000]) and three cold-repressed differential genes (AtDTX29, XTH1, and TLP) were screened.

## Graphical abstract

**Figure:**
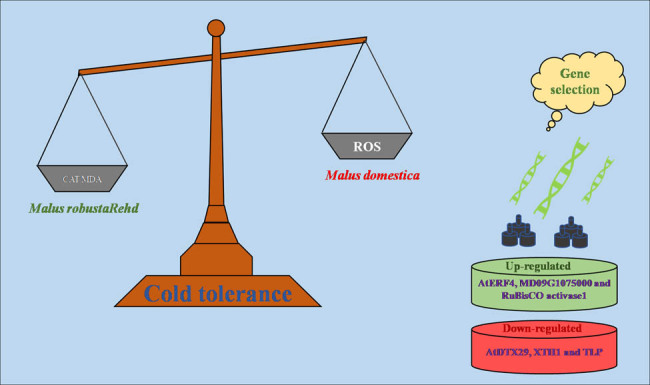

## 1 Introduction

With the intensification of global climate change, the trend of warming in winter and early spring in apple-producing areas represented by China in the northern hemisphere is gradually increasing [1,2]. It is manifested with the increase in warm winter and warm spring years, and the increase in inter-annual temperature fluctuations, which leads to the advancement of fruit tree phenology [3]. However, as the temperature rises faster in spring, it is prone to a drastic cooling process, which increases the risk of fruit trees suffering from late frost during the flowering period [4]. The damage of chills to flowers and early-developing fruits is one of the biggest limiting factors in fruit tree growing areas in the world [5,6]. Particularly, the screening of cold-resistant genes in trees is an important choice for cultivating cold-resistant varieties. Therefore, breeding cold-tolerant apple varieties is one of the important measures to reduce the freezing damage during the flowering period of apples, but there are few research studies on the function of cold-resistant genes during the flowering period.

There are a series of complex response mechanisms of physiological and biochemical changes in plants to adapt to low temperature and improve cold resistance to adapt to the low-temperature environment. This is called cold adaptation. In the process of cold adaptation, low temperature stimulates the down-regulation of active species (reactive oxygen species [ROS], malondialdehyde [MDA], superoxide dismutase [SOD], and peroxidase) [7,8], changes in signal substances such as ABAaecium, etc., through receptor proteins, and protein kinases on the cell membrane transduce these signals, activate the expression of a large number of transcription factor genes and cold-responsive protein genes, improve the antioxidant capacity and osmotic regulation ability, promote the reconstruction of the cell membrane system, and the material balance inside and outside the membrane to adapt to the low-temperature environment [9–14]. Flowers under cold stress are prone to withering and abnormal softening. Softening is a complex process, and changes in the composition of the cell wall and the external encapsulation structure are the key factors leading to softening. This process involves the synergy of a series of cell wall modification enzymes, most of which are hydrolases, and are directly related to abnormal changes in cell wall metabolism [15]. Cell wall modification is closely related to the activities of polygalacturonate, cellulase, xyloglucan endo-glycosyltransferase/hydrolase, and other related enzymes [16,17].

With the development of genome sequencing technology, it is necessary to further understand the activation and silencing of genes as low temperatures proceed. In order to quickly sense and respond to cold, plants have evolved a series of cold signaling pathways. Among them, the cold response genes (CORs) are the key in the activation pathway [9]. It is reported that the main regulatory cascade of plant cold response is composed of C-repeat binding factors (CBFs), which are involved in cold adaptation [18]. Therefore, one of the keys to promote the cultivation of frost-resistant apple is to find the key genes regulating CBFs. To study the chilling tolerance characteristics of different varieties of apple flowers, a lot of research has been invested and results have been obtained [19–23]. However, research on the genetic level is still insufficient.

To this end, we set up two apple tissue treatments (*Malus robusta* Rehd and *Malus domestica*) and three low-temperature gradients (0, −2, and −4°C), and performed a transcriptome analysis on apple flower tissue samples. Although, previous studies have determined that *M. robusta* Rehd flowers are relatively resistant to cold, while *M. domestica* flowers are relatively weak, in-depth gene-level explanations are still lacking. To make up for this defect, the purpose of this article is to compare and analyze the genomes of apples under different temperature gradients through transcriptome sequencing. At the genomic level, some important functional cold tolerance genes and related signaling pathways were identified and validated by quantitative real-time reverse transcriptase PCR (qRT-PCR). This study revealed potential cold-resistant genes and associated signal pathways through the perspective of genomics, laying a foundation for the subsequent functional studies of a certain cold-resistant gene.

## 2 Materials and methods

### 2.1 Preparation of apple blossom tissue samples

Herein, in order to study the resistance of different varieties of apple flowers to cold and their genomics evidence. We set up two varieties of apple flower groups, group A (*M. robusta* Rehd) and group B (*M. domestica*) each with three low-temperature gradient treatments (0, −2, and −4°C), and a room temperature of 25°C as control. In short, the sample preparation procedures are as follows: (1) Randomly select three trees of same age and tree vigor of two varieties in the apple resource garden, and wait until the third day after each variety reaches the full flowering stage: group A (*M. robusta* Rehd) and group B (*M. domestica*). (2) On the main branch at two-third of the height of each tree from the ground, select one 2-year-old flowered branch from each of the four vertical directions on the horizontal. (3) After standing at room temperature (25 ± 1°C) for 2 h, put the two varieties into the high and low-temperature (alternating) test box (Shanghai Yiheng Technology Co., Ltd), and set four temperature gradients, 0, −2, −4°C, and a room temperature control group. (4) Then, after refrigerating for 2 h at each temperature gradient, flowers were taken for transcriptome and related enzyme activity detection. The sample names are set as A0, A2, A4, and AN; B0, B2, B4, and BN (the numbers 0, 2, 4, and N correspond to 0, 2, 4, and 25°C temperature treatment groups, respectively). Tissue samples of apple flowers were collected after 2 h of treatment, and transcriptome analysis was performed after quick freezing in liquid nitrogen. *M. robusta* Rehd and *M. domestica* were provided by the demonstration base of apple cultivation technology from the Institute of Horticulture, Ningxia Academy of Agriculture and Forestry Sciences, Yinchuan, Ningxia, China.

### 2.2 Antioxidant test

The determination of ROS, SOD, MDA, and catalase (CAT) is carried out strictly according to the manufacturer’s instructions. Herein, ROS (Commodity number: E004-1-1), SOD (Commodity number: A001-3-2), MDA (Commodity number: A003-1-2), and CAT (Commodity number: A007-1-1) are all measured with kits, which are produced by Nanjing Jiancheng Biotechnology Co., Ltd. Any operation shall be carried out in strict accordance with the manufacturer’s instructions. Unless otherwise specified, other reagents used are of analytical grade, and the water used in the experiment is deionized water (18.2 MΩ). The apple blossoms treated with temperature for 2 h were collected, and the cells were disrupted at 4°C for homogenization.To obtain the tissue homogenate, place 1 mL in a centrifuge tube, centrifuge at 4,200 rpm for 6 min, and remove the supernatant. Later sterile PBS is added to the pellet to wash once and then centrifuge it. Subsequently, processing and determination were performed according to the method of the kit.

### 2.3 RNA extraction and transcriptome sequencing

The extraction of total RNA from apple flowers was carried out according to previous methods [24]. In short, TRIzol reagent (Invitrogen, USA) was used to isolate total RNA in each group of apple blossom samples for subsequent transcriptome analysis. Note that in the transcriptome test processing, each sample uses nine biological replicates, and the total RNA obtained from every three replicates is combined to obtain a total of three biological replicate samples. Each sample used 5 μg RNA samples to construct a transcriptome library. The cDNA library was constructed by Shanghai Meiji Biomedical Technology Co., Ltd (www.majorbio.com, Shanghai, China) using the Illumina HiSeq platform (Illumina Inc., San Diego, CA, USA) for sequencing. After the library is constructed, use the Qubit2.0 Fluorometer for preliminary quantification, dilute the library to 1.5 ng/μL, and then use the Agilent 2100 bioanalyzer to detect the insert size of the library. After the insert size meets expectations, qRT-PCR measures the effective concentration of the library. Accurate quantification [25] (the effective concentration of the library is higher than 2 nM) to ensure the quality of the library. After a quick filter, use HISAT 2.2.4 to compare the clean reads with the apple genome (https://iris.angers.inra.fr/gddh13/index.html). For each transcribed region, use the RESM software to calculate the number of fragments per kilobase transcript in the mapped reads per million (FPKM) value. DESeq2 software is used to identify differentially expressed genes (DEGs) by fold change ≥2 and divergence probability ≥0.8. Use gene ontology (GO) and Kyoto Encyclopedia of genes and genomes (KEGG) tools to analyze DEG.

### 2.4 qRT-PCR verifies high expression genes for cold tolerance

Screen the potentially highly expressed genes during the cold treatment process by the number of counts, and select the first three of them for qRT-PCR verification. The qRT-PCR method will not be repeated [26,27], and the primary design is based on the gene ID with reference to the NCBI database (https://www.ncbi.nlm.nih.gov) [28,29]. The Revert Aid First Strand cDNA Synthesis Kit (Thermo Scientific, Waltham, MA, USA) is used to perform qRT-PCR. To standardize the different genes in the cDNA samples, the gene elongation factor 1α (EF-1α; DQ341381) in apples was used. The 2^−ΔΔCT^ method is used to calculate the relative expression level of each gene. Three biological samples were used in all experiments.

### 2.5 Statistical analysis

Unless otherwise specified, all experiments were performed in triplicate (*n* = 3). IBM SPSS 22.0 software was used to perform statistical analysis on the data from the control and different low-temperature treatments through analysis of variance (one-way analysis of variance). The probability value of *p* < 0.05 is considered to indicate a statistically significant difference and is indicated by *. All data are expressed as the mean ± standard deviation of three replicates.

## 3 Results and discussion

### 3.1 Low temperature induces anti-oxidative stress

**Figure 1:**
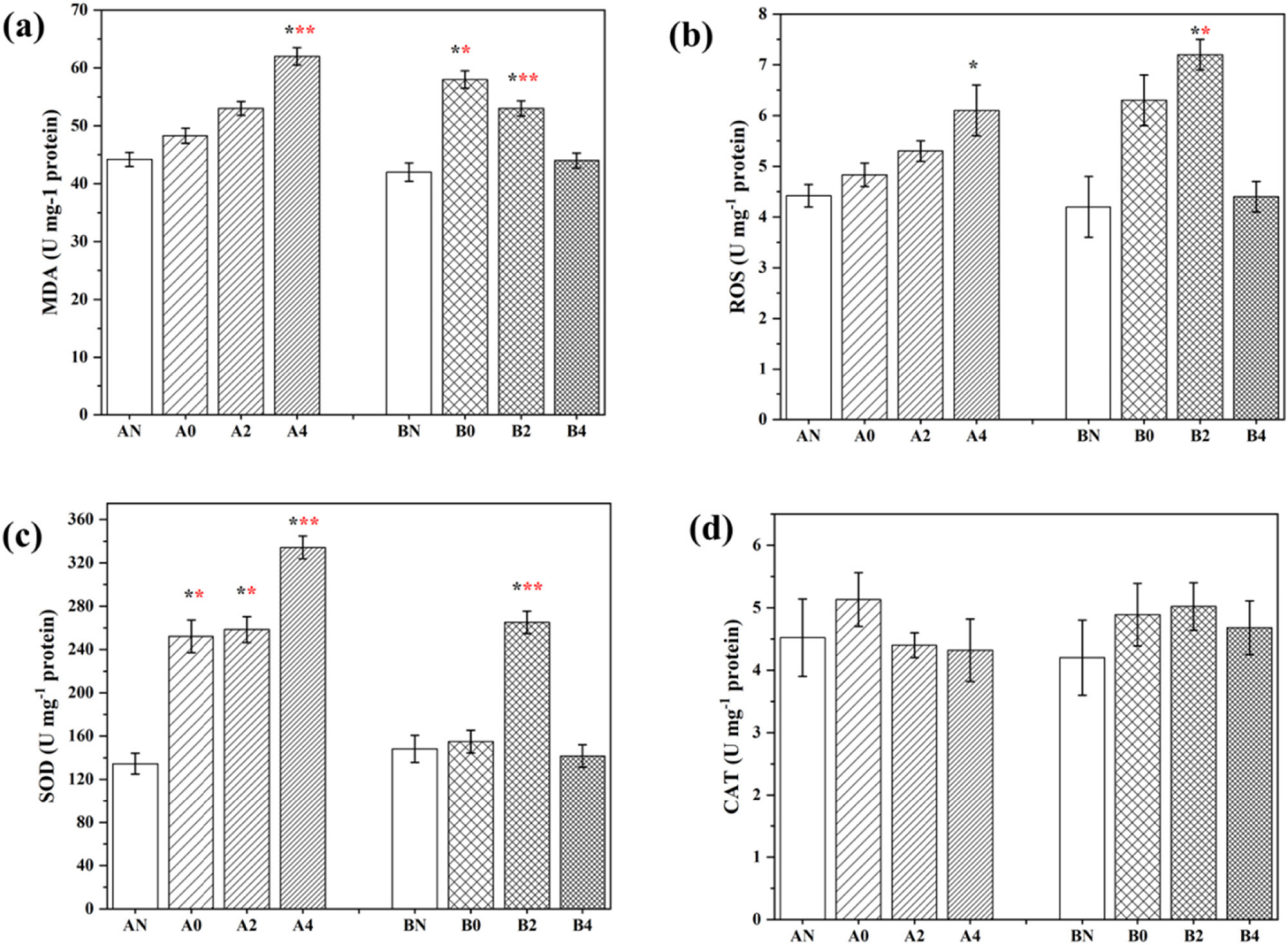
Antioxidant test of apple flower cells after low-temperature treatment: (a) MDA enzyme content, (b) ROS content, (c) SOD content, and (d) CAT content. Statistical differences: *, **, *** represent the results with *p* values less than 0.05, 0.005, and 0.001 (compared to their respective normal groups).

In order to test the oxidative stress levels in the cells of two varieties of apple flowers after low temperature and cold treatment, we tested the activity of ROS, SOD, MDA, and CAT [30]. Herein, we found that low-temperature cold treatment can significantly cause the production of oxidative stress species such as ROS and MDA. In this regard, the samples of group A showed obvious temperature dependence. Low-temperature treatment at −4°C can significantly increase the production of such strong oxides (*p* < 0.001); at the same time, the expression of antioxidant enzyme SOD is also temperature dependent (the expression is up-regulated as the temperature decreases). However, although group B has cold stress-induced oxidative stress and over-release of antioxidant enzymes, it does not have a strong correlation similar to temperature dependence in group A. As far as the elimination of hydrogen peroxide is concerned, the expression of CAT enzyme has no obvious difference. The above results are shown in Figure 1.

### 3.2 Statistics of second-generation sequencing results

Through genomics identification, the number of clean reads with an error rate of less than 0.03% accounted for 97.52–98.53% of all data, and the Q30 value of all samples was 92.74–94.04% (Tables S1 and S2; Figures S1 and S2).

### 3.3 Quantitative analysis of second-generation sequencing results and screening of differential genes

**Figure 2:**
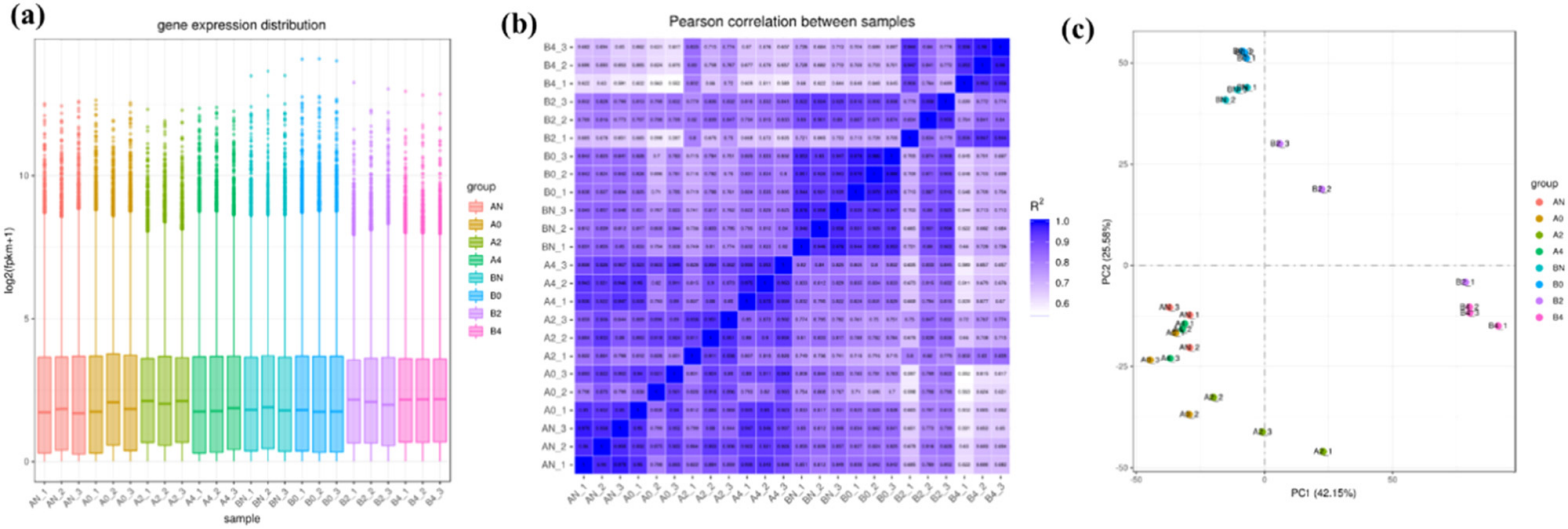
Transcriptome test quantitative analysis results: (a) gene expression distribution, (b) correlation between samples, and (c) principal component analysis (species, temperature, and treatment).

The screening criteria for differential genes are very important. Here we give the criteria |log2(Foldchange)| ≥ 1&padj ≤ 0.05 are commonly used empirical values [31]. For the obtained sequencing results, we conducted a statistical analysis. In particular, we screened the first three high-expressed differential genes and the first three low-expressed genes. In Tables S3 and S4 and Figure S3, the ID and count of the three genes are given to make a histogram for visualization. The results showed that AtDTX29 (ID: MD16G1102700), XTH1 (ID: MD13G1134600), and TLP (ID: MD04G1064200) genes were down-regulated in the samples of groups A and B; AtERF4 (ID: MD03G1231800), MD09G1075000 (name unknown), and RuBisCO activase 1 (ID: MD11G1095300) genes. Moreover, as the temperature changes, the expression of the six genes in group A is obviously temperature dependent; in contrast, group B does not show a regular trend. Due to the influence of sequencing depth and gene length, the gene expression value of RNA-seq is generally not expressed by read count, but by FPKM. FPKM corrects sequencing depth and gene length successively to compare and evaluate gene expression distribution [32]. Statistics on gene expression distribution show that the gene expression distribution of various apple flower samples is relatively stable (Figure 2a). The results showed that the log2 (FPKM + 1) value was relatively stable, concentrated in 1.72–1.83. It indicated that the gene distribution was relatively balanced and the quantitative results were good [33]. In addition, the correlation between the two apple blossom samples under low-temperature treatment was compared and analyzed (Figure 2b). The results show that low-temperature treatment will significantly change the biological correlation between the samples of group B, and this change is not temperature dependent. Group A showed temperature-dependent differences, and as the temperature decreased, the biological relevance became worse. Similarly, the results of this correlation analysis are similar, low-temperature treatment will significantly change the biological correlation between the samples of group B, and this change is not temperature dependent. Group A showed temperature-dependent differences, and as the temperature decreased, the biological relevance became worse. In addition, the principal component analysis showed that the species difference explained 42.15% of the difference between the two groups of apple samples; temperature treatment explained 25.58% of the difference (Figure 2c).

**Figure 3:**
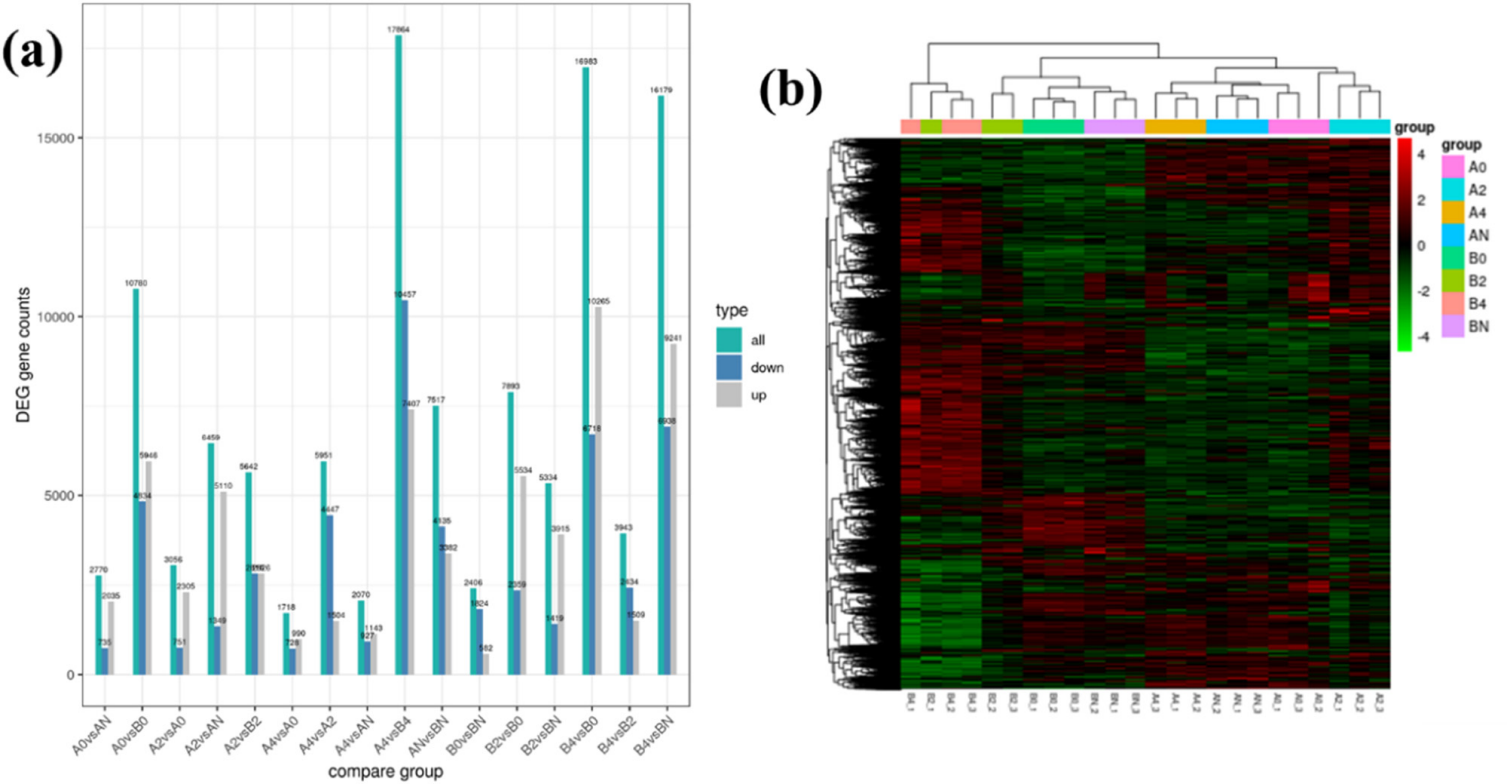
Analysis of the difference in sequencing results between different apple varieties and treatment temperature groups: (a) differential gene statistics (all, the number of up-regulated and down-regulated genes) and (b) differential gene clustering.

In addition, we also statistically compared the number of different genes between each group, up and down, respectively, representing the number of up-regulated and down-regulated genes (Figure 3a). This figure can be used to preliminarily judge the difference in gene expression between samples of each group. It can be seen from the figure that B4 and A4, BN and B4, B0 and B4 have the most DEGs, which is consistent with the previous results (showing higher biological differences). The heat map also shows the same result (Figure 3b).

### 3.4 Enrichment analysis

**Figure 4:**
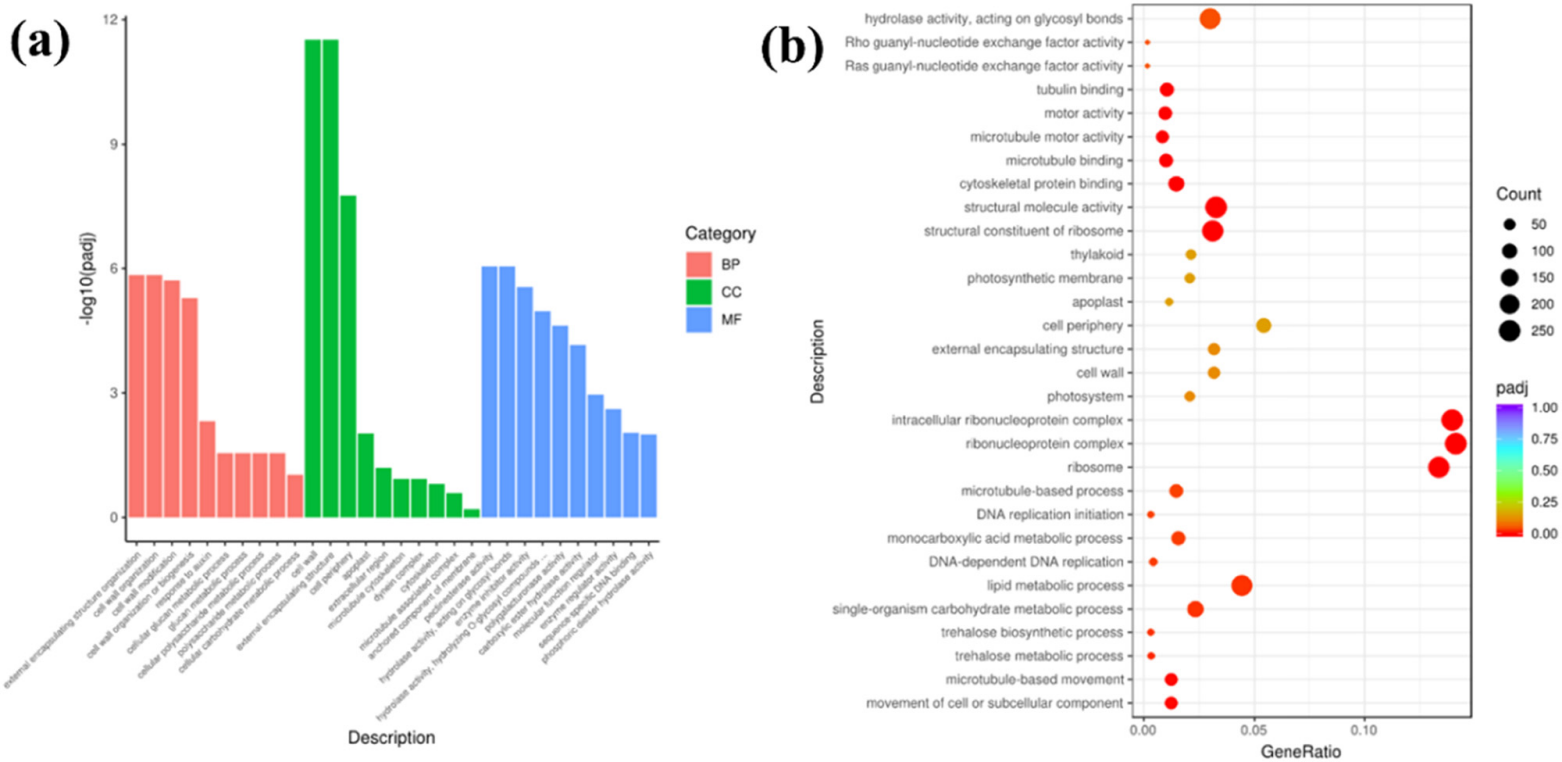
GO enrichment analysis: (a) GO enrichment analysis histogram and (b) GO enrichment analysis scatter plot (A4 vs B4).

**Figure 5:**
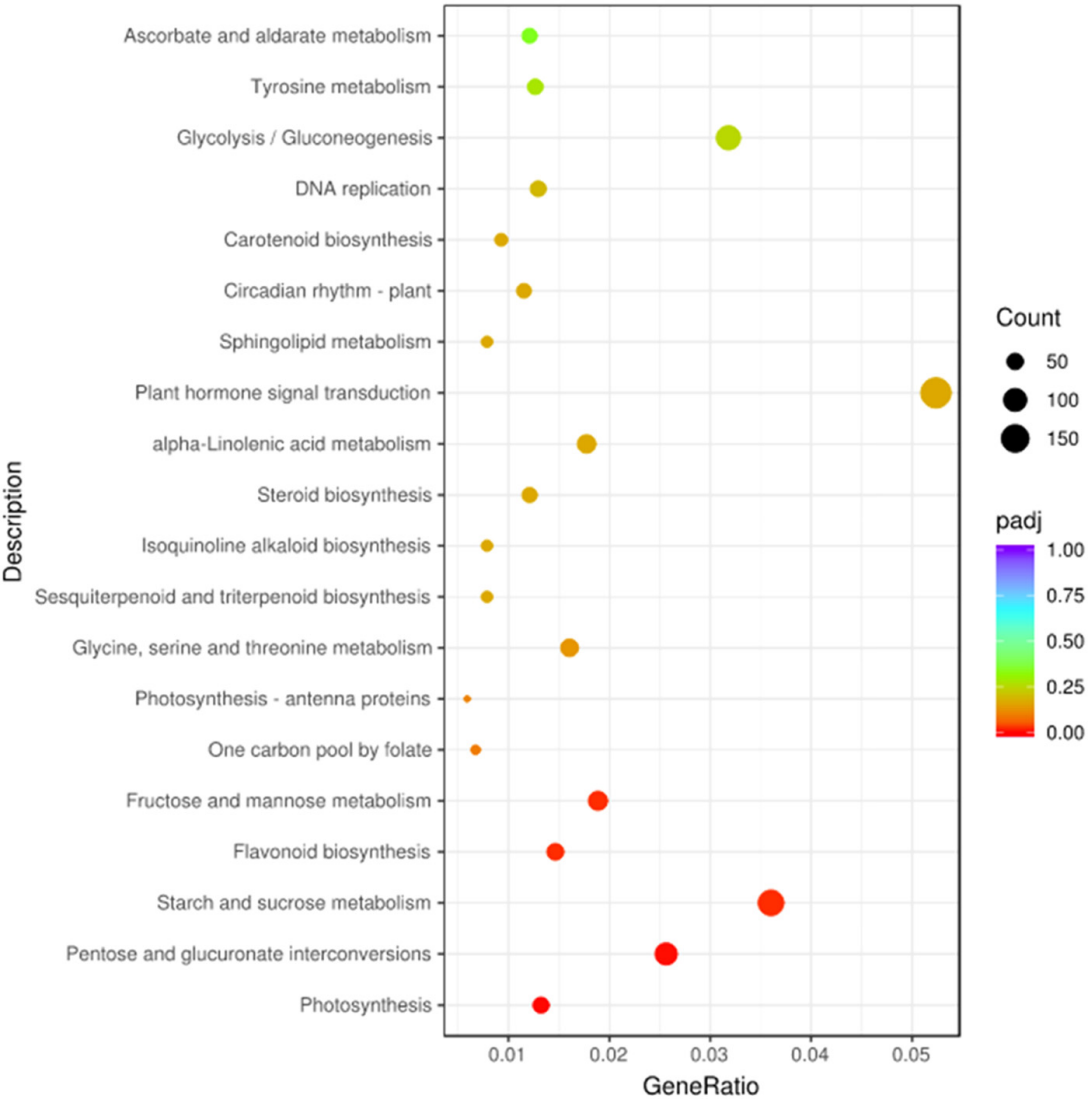
KEGG signal pathway analysis scatter diagram.

The enrichment analysis of the two apple flower gene functions under different temperature treatments is expected to discover the biological pathways that play a key role in the biological process, so as to reveal and understand the basic molecular mechanism of the biological process [34,35]. First, we analyzed the GO of the overall sample (Figure S4).The GO enrichment of the overall sample found that: (1) biological processes are mostly concentrated in signal-organism process, metabolic process, cellular process, and biological regulation process; (2) cytological processes are mostly concentrated in cell, cell part, membrane, membrane part, organelle, organelle part, etc.; and (3) the molecular process focuses on binding and catalytic activity. The GO analysis of the overall sample shows that low-temperature treatment can induce fluctuations in cell structure, membrane structure, metabolism, organic molecules, enzyme activity, and catalytic processes [36]. In order to further analyze the internal differences, we further performed enrichment analysis on A4 and B4. The GO analysis results show that in the face of low temperature and cold damage: (1) biological function: external encapsulation structure organization, cell wall, and biogenesis, cell wall modification is the main functional response; (2) cytological function: cell wall, external packaging tissue, and cell periphery are the main functional response; and (3) at the molecular level: pectin esterase activity, hydrolase activity, enzyme inhibitor activity, and polygalacturonate activity are the main response. The GO enrichment analysis of B4 and A4 found that the main differences are the hydrolysis of O-glycosyl, the action on the glycosyl bond, the activity of structural molecules, ribosomes, lipid metabolism, and sugar metabolism pathways (Figure 4). The results show that the metabolism of carbohydrates and the expression of related hydrolytic enzymes are the main reasons for the biological differences between the two samples. KEGG results showed that plant hormone signal transduction, starch and sucrose metabolism, the interaction of pentose and glucuronic acid, and glycolysis/gluconeogenesis are the main differentials signaling pathways between B4 and A4 (Figure 5).

### 3.5 qRT-PCR verification to confirm chilling resistance gene

**Figure 6:**
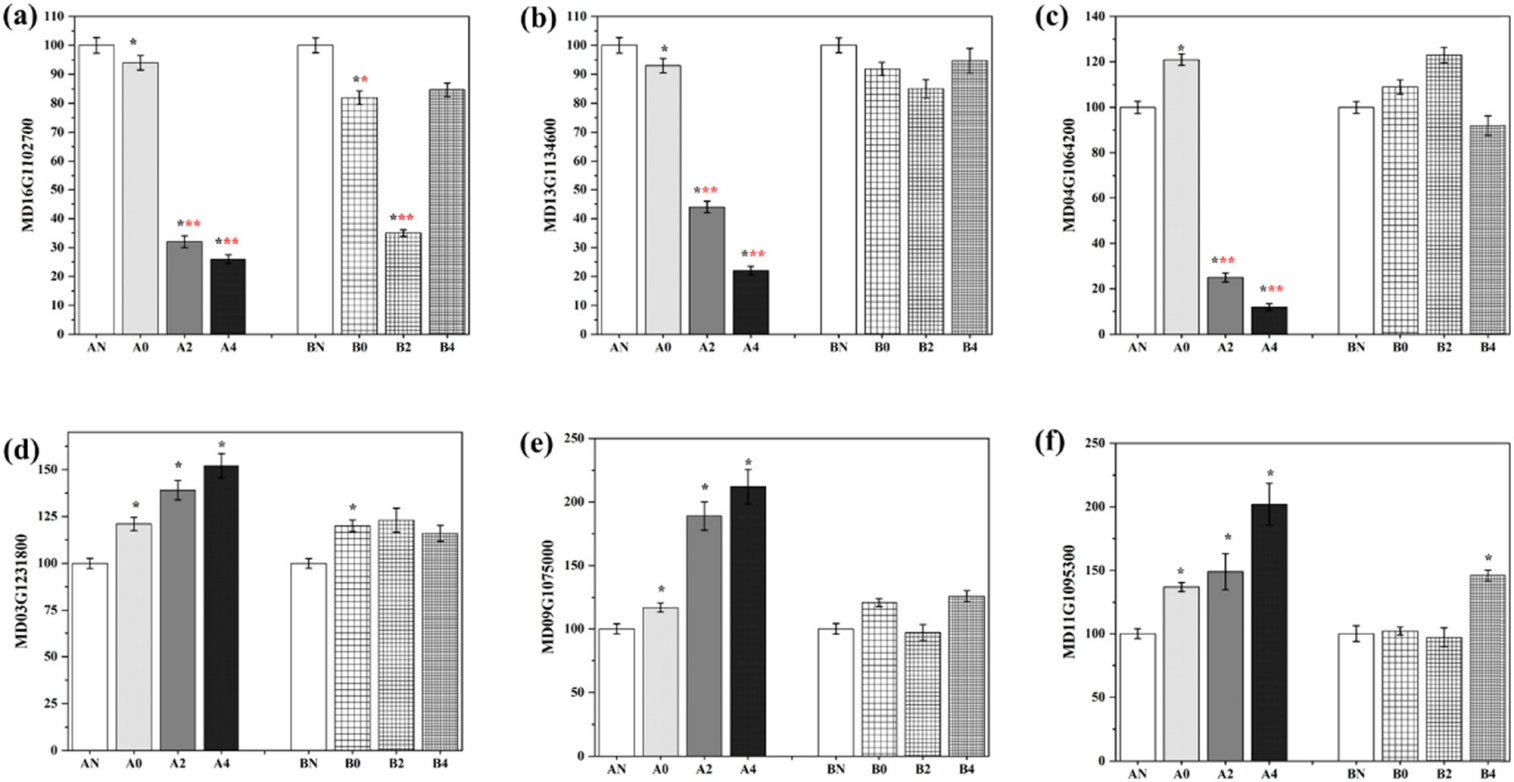
qRT-PCR verification of the first three DEGs: (a–c) the first three genes whose expression is down-regulated and (d–f) the first three genes whose expression is up-regulated. (a) MD16G1102700, (b) MD13G1134600, (c) MD04G1064200, (d) MD03G1231800, (e) MD09G1075000, and (f) MD11G1095300. Statistical differences: *, **, *** represent the results with *p* values less than 0.05, 0.005, and 0.001 (yellow and blue are compared to their respective normal groups).

This study found six differential genes; three differential genes suppressed by cold (AtDTX29, XTH1, and TLP); three potential cold-tolerant genes AtERF4, MD09G1075000 (name unknown), and RuBisCO activase 1. To this end, we provide the qRT-PCR method for verification. The results showed that the expression of AtDTX29, XTH1, and TLP genes in group A samples was down-regulated (Figure 6a–c). Among them, the expression difference in the −4°C treatment group was the most significant (*p* < 0.001), which was consistent with the transcriptome test results (Figure S3). For group B, there is no regular explanation. The same up-regulated AtERF4, MD09G1075000 (name unknown), and RuBisCO activase 1 genes were also verified. The expressions of the three genes in group A were all up-regulated.

## 4 Discussion

Freezing stress at flowering stage was one of the main limiting factors for apple yield and quality [37]. Apple’s response to low temperatures is a complex pathway involving many CORs. Therefore, the information of failure critical CORs is of great significance for the screening and breeding of hardy varieties. In this study, we found that AtDTX29, XTH1, TLP, AtERF4, MD09G1075000 (name unknown), and RuBisCO activase 1 genes were crucial to the cold tolerance of apple, which provided support for the breeding of cold-resistant apple varieties.

In recent years, scholars have done a lot of research work on the relationship between enzymes and the cold resistance of fruit trees. It mainly uses apples, oranges, tomatoes, and grapes as the test materials, and points out ATPase, peroxidase, amylase, invertase, cheese dehydrogenase, phospholipase, glutathione reductase, etc. The differences in the expression of these enzymes or isoenzymes have a certain relationship with the cold resistance of fruit trees [38,39]. Chilling damage can cause the rupture of some organelle membranes inside the cell, and induce oxidative stress [40,41]. Meanwhile, under cold stress, the cell’s enzyme digestion activity is enhanced, i.e., to resist cold invasion by increasing the release of peroxide [42]. It was found that cold exercise on apples can cause changes in the activity of antioxidant enzymes. In this work, under low-temperature stress, both groups A and B detected up-regulated expression of active species. However, in the face of the same oxidative stress, as the expression of oxides in group A increases, the release of oxide scavenging enzymes in the body can be promoted, which can alleviate the accumulation of active substances. In contrast, samples of group B can also increase the expression of scavenging enzymes. But it is inconsistent with the expression of oxidant species, instead, it makes the level of active species in the cell disordered and affects normal cell functions. Moreover, in the face of low-temperature stress from 0 to −4°C, the temperature-dependent changes in the expression of group A antioxidant enzymes can effectively inhibit the excessive accumulation of active species in the cell. Plants can resist ROS stress and maintain ROS dynamic balance by regulating the expression of antioxidant enzymes to varying degrees [39]. This research study believes that group A (*M. robusta* Rehd) apple flower samples are significantly stronger than group B (*M. domestica*) in anti-oxidative stress when faced with low-temperature stress.

Apple flowers in group A showed obvious biological differences in the face of low-temperature treatment, which was attributed to the expression of more and more differential genes; group B did not have similar conclusions. On the contrary, the gap between A4 and AN is smaller (Figure 3). The interspecies difference between group A and group B was the most significant in the −4°C treatment group. In the process of resisting the cold, we might as well guess that group B (*M. domestica*) apple flowers cannot adapt to low temperatures or can adapt to low temperatures in a small range. Group A (*M. robusta* Rehd) apple flowers can regulate their own genetic difference expressions to respond to temperature changes, and this mechanism is relatively perfect, so that group A can adapt to more severe temperature disturbances. The research of László et al. proved this view [43]. Moreover, to prove our conjecture, enrichment analysis and qRT-PCR were used in the subsequent design.

Enrichment analysis showed that the main differences between groups A and B in low-temperature adaptation are the differences in enzyme activity expression, cell wall and cell membrane structure, glucose metabolism/gluconeogenesis, and signal transmission processes (Figure 4). The possible explanation is that group A cells undergo a series of complex changes in the face of low-temperature stress. First, in the face of low-temperature stress, group A apple flowers can quickly receive this information through the receptors; next, ribosomes coordinate gene expression to produce special proteins (enzymes, signal molecules, etc.) [39,44–47], which act on cell wall/membrane formation and cell metabolism of internal energy storage species (carbohydrates) to resist chilling stress. In contrast, group B apple flowers cannot coordinately mobilize the expression of a series of related genes to resist the cold. More and more evidence show that the response of plants to abiotic stress is accompanied by extensive changes in the cell wall, including the content and structure of cell wall polysaccharides, cell wall modifying enzymes, and cell wall hardness [48]. Cycle is the central biochemical pathway of cell metabolism. It has two main functions: synthesis of precursors and generation of energy. Many genes involved in cell wall biosynthesis, assembly, or modification have been shown to play a key role in abiotic stress tolerance [49]. The plant body in group A enhances cold tolerance by affecting the characteristics of the apple flower cell wall. TCA cycle is the central biochemical pathway of cell metabolism [50]. It has two main functions: synthesis of precursors and generation of energy. Herein, the enrichment analysis found that group A of apple flower plant cells enhanced glycolysis/gluconeogenesis and carbohydrate cell metabolism to provide the body with sufficient energy to address cold.

This study identified six differential genes; three differential genes suppressed by cold (AtDTX29, XTH1, and TLP); three potential cold tolerance genes (AtERF4, MD09G1075000 [name unknown], and RuBisCO activase 1). The primary plant cell wall consists of cellulose microfibers and matrix polysaccharides in which xyloglucan forms a backbone network and strengthens the rigidity of the cell wall by forming hydrogen bonds with cellulose fibril [51]. Xyloglucan metabolism is regulated by xyloglucan glycosylase/hydrolase (XTH), which plays a key role in fruit ripening by loosening the cell wall and preparing for further modification of other cell wall-related enzymes [52]. Somalin-like protein (TLP) has been reported to have antifreeze activity, and its potential role as an antifreeze substance has been demonstrated in plants [53]. Herein, the up-regulation of XTH1 and TLP expression showed the response of flowering apples to cold. Studies have shown that the gene AtERF4 encodes a transcription factor regulated by low temperature and ethylene [54]. In addition, the AtERF4 protein complex inhibits abscisic acid and abiotic stress responses through deacetylation modification [55]. RuBisCo activity is regulated by Rubisco activating enzyme (RCA). RCA prevents the inhibition of Rubisco sites by promoting the ATP-dependent conformational changes that open the closed site, thereby making them more accessible to solvents and promoting the dissociation of inhibitory sugar phosphates [56]. During the entire cold acclimation (CA) process, the short isoform RCA enzyme of the perennial ryegrass (*Lolium perenne*, Lp) highly frost-tolerant genotype has a higher abundance, and at the same time, the accumulation of CA after CA decreases in the low-frost-tolerant genotype [57]. Among them, the difference in the −4°C group was the largest and had a significant difference (*p* < 0.05). Similar to the results of the enrichment analysis, the cold-resistant variety A enhances its cold-resistant characteristics by enhancing the composition and structure of the cell wall and adjusting the expression of related genes. AtERF genes are affected by ethylene and abiotic stress conditions (such as injury, cold, high salinity, or drought), differential regulation, through ETHYLENE-INSENSITIVE2 (EIN2) dependent or independent pathway, according to this to resist chills [58,59]. Therefore, AtERF4, MD09G1075000 (name unknown), and RuBisCO activase 1 are considered as potential cold hardiness genes.

## 5 Conclusion

In summary, the oxidase and antioxidant activity tests showed that *M. robusta* Rehd (group A) showed a more favorable ROS clearance than *M. domestica* (group B). In addition, differential gene analysis showed that *M. robusta* Rehd produced more differential genes in the face of cold. *M. robusta* Rehd and *M. domestica* produced differences in enzyme activity expression, cell wall and cell membrane structure, glucose metabolism/gluconeogenesis, and signaling processes when faced with the cold. Finally, six potential cold-resistant genes AtDTX29, XTH1, TLP, AtERF4, MD09G1075000 (name unknown), and RuBisCO activase 1 were detected and verified. This work has shown that *M. robusta* Rehd has better cold resistance. It is expected that through the regulation of expression or gene programming of potential cold-resistant genes, we will further screen out good varieties with excellent cold resistance.

## Supplementary Material

Supplementary material
