# Supplementary material for: Comparative transcriptomes reveal molecular mechanisms of apple blossoms of different tolerance genotypes to chilling injury
Source: Open Life Sci. 2023 Dec 28;18(1):20220613. doi: 10.1515/biol-2022-0613 (PMC10756277; doi:10.1515/biol-2022-0613)
Supplement: Supplementary material [file biol-2022-0613-sm.pdf]

Table S1. Summary of sample sequencing data quality

Sample	Raw reads	Clean reads	Error rate(%)	Q20 (%)	Q30 (%)	GC (%)
A0_1	42201982	41160058	0.03	97.79	93.39	47.57
A0_2	44780628	43822204	0.03	97.68	93.15	47.48
A0_3	42074838	40967762	0.03	97.97	93.79	47.72
A2_1	47033172	45998720	0.03	97.84	93.44	46.51
A2_2	48253210	47321564	0.03	97.77	93.26	46.57
A2_3	45505576	44506126	0.03	97.81	93.38	46.53
A4_1	44952624	44099498	0.03	97.73	93.16	46.86
A4_2	48262840	47307414	0.03	97.84	93.39	46.9
A4_3	49567610	48614324	0.03	97.75	93.23	46.92
AN_1	43673018	42589912	0.03	97.97	93.75	46.74
AN_2	42003616	41095898	0.03	97.9	93.59	46.72
AN_3	48494194	47531448	0.03	97.77	93.33	47.14
B0_1	44477498	43532594	0.03	97.9	93.61	46.85
B0_2	49136504	48005368	0.03	97.94	93.67	46.9
B0_3	47759384	47003834	0.03	97.93	93.63	46.82
B2_1	49699990	48923604	0.03	97.56	92.74	46.65
B2_2	49770820	48896420	0.03	97.62	92.95	46.9
B2_3	43216822	42466108	0.03	97.92	93.62	46.79
B4_1	45240068	44451370	0.03	97.93	93.66	47.02
B4_2	43596952	42724340	0.03	97.71	93.16	47.03
B4_3	46387840	45436794	0.03	97.99	93.79	47.04
BN_1	43605762	42851398	0.03	97.96	93.69	46.89
BN_2	47333066	46317896	0.03	98.01	93.88	46.84
BN_3	47276760	46268788	0.03	98.05	94.04	46.86

Table S2. Statistics of sample and reference genome comparison

sample	total_reads	total_map	unique_map	multi_map	read1_map	read2_map	positive_map	negative_map	splice_map	unsplice_map	proper_map
A0_1	41160058	37336647(90.71%)	36577409(88.87%)	759238(1.84%)	18376751(44.65%)	18200658(44.22%)	18281176(44.41%)	18296233(44.45%)	12856271(31.23%)	23721138(57.63%)	34125792(82.91%)
A0_2	43822204	39577711(90.31%)	38714825(88.35%)	862886(1.97%)	19466104(44.42%)	19248721(43.92%)	19331431(44.11%)	19383394(44.23%)	13558600(30.94%)	25156225(57.41%)	35939350(82.01%)
A0_3	40967762	37120628(90.61%)	36384293(88.81%)	736335(1.8%)	18252098(44.55%)	18132195(44.26%)	18177578(44.37%)	18206715(44.44%)	12540686(30.61%)	23843607(58.2%)	33980084(82.94%)
A2_1	45998720	41209761(89.59%)	40266852(87.54%)	942909(2.05%)	20217110(43.95%)	20049742(43.59%)	20118824(43.74%)	20148028(43.8%)	14299430(31.09%)	25967422(56.45%)	37533046(81.6%)
A2_2	47321564	42555473(89.93%)	41672717(88.06%)	882756(1.87%)	20947028(44.27%)	20725689(43.8%)	20822767(44.0%)	20849950(44.06%)	14520328(30.68%)	27152389(57.38%)	38858578(82.12%)
A2_3	44506126	39893904(89.64%)	39026771(87.69%)	867133(1.95%)	19605273(44.05%)	19421498(43.64%)	19494842(43.8%)	19531929(43.89%)	13591476(30.54%)	25435295(57.15%)	36550776(82.13%)

A4_1	44099498	39963594(90.62%)	39144011(88.76%)	819583(1.86%)	19674329(44.61%)	19469682(44.15%)	19568996(44.37%)	19575015(44.39%)	14102380(31.98%)	25041631(56.78%)	36609140(83.01%)
A4_2	47307414	42944430(90.78%)	42088727(88.97%)	855703(1.81%)	21137363(44.68%)	20951364(44.29%)	21040397(44.48%)	21048330(44.49%)	15070758(31.86%)	27017969(57.11%)	39441038(83.37%)
A4_3	48614324	44007203(90.52%)	43090799(88.64%)	916404(1.89%)	21649792(44.53%)	21441007(44.1%)	21533528(44.29%)	21557271(44.34%)	15375525(31.63%)	27715274(57.01%)	40307808(82.91%)
AN_1	42589912	38665646(90.79%)	37883904(88.95%)	781742(1.84%)	19002750(44.62%)	18881154(44.33%)	18938018(44.47%)	18945886(44.48%)	13867710(32.56%)	24016194(56.39%)	35636160(83.67%)
AN_2	41095898	37144904(90.39%)	36395887(88.56%)	749017(1.82%)	18269869(44.46%)	18126018(44.11%)	18191451(44.27%)	18204436(44.3%)	13139920(31.97%)	23255967(56.59%)	33747070(82.12%)
AN_3	47531448	43088931(90.65%)	42198025(88.78%)	890906(1.87%)	21192515(44.59%)	21005510(44.19%)	21095668(44.38%)	21102357(44.4%)	15242177(32.07%)	26955848(56.71%)	39376558(82.84%)
B0_1	43532594	40451488(92.92%)	39590692(90.94%)	860796(1.98%)	19863499(45.63%)	19727193(45.32%)	19783477(45.45%)	19807215(45.5%)	13504131(31.02%)	26086561(59.92%)	37732298(86.68%)
B	48	4464	4372	919	2193	2179	2184	2187	1496	2875	4176

0	00	1762	2385	377(	0411	1974	8349	4036	6903	5482	7514
_	53	(92.9	(91.0	1.92	(45.6	(45.3	(45.5	(45.5	(31.1	(59.9	(87.0
2	68	9%)	8%)	%)	8%)	9%)	1%)	7%)	8%)	%)	1%)
B	47	4373	4279	942	2146	2133	2138	2140	1483	2795	4081
0	00	4369	2174	195(	1733	0441	8459	3715	7486	4688	5202
_	38	(93.0	(91.0	2.0	(45.6	(45.3	(45.5	(45.5	(31.5	(59.4	(86.8
3	34	4%)	4%)	%)	6%)	8%)	%)	4%)	7%)	7%)	3%)
B	48	4538	4427	1104	2227	2200	2211	2215	1637	2790	4175
2	92	2626	7692	934(	1866	5826	9981	7711	7070	0622	2452
_	36	(92.7	(90.5	2.26	(45.5	(44.9	(45.2	(45.2	(33.4	(57.0	(85.3
1	04	6%)	%)	%)	2%)	8%)	1%)	9%)	7%)	3%)	4%)
B	48	4546	4447	982	2235	2211	2222	2225	1596	2851	4215
2	89	0217	8027	190(	9252	8775	3823	4204	6564	1463	3644
_	64	(92.9	(90.9	2.01	(45.7	(45.2	(45.4	(45.5	(32.6	(58.3	(86.2
2	20	7%)	6%)	%)	3%)	4%)	5%)	1%)	5%)	1%)	1%)
B	42	3956	3872	842	1942	1929	1934	1937	1364	2507	3689
2	46	5751	3013	738(	4570	8443	9884	3129	9989	3024	1364
_	61	(93.1	(91.1	1.98	(45.7	(45.4	(45.5	(45.6	(32.1	(59.0	(86.8
3	08	7%)	9%)	%)	4%)	4%)	7%)	2%)	4%)	4%)	7%)
B	44	4131	4035	961	2024	2010	2016	2019	1465	2570	3850
4	45	8609	6822	787(	7863	8959	0807	6015	2092	4730	1880
_	13	(92.9	(90.7	2.16	(45.5	(45.2	(45.3	(45.4	(32.9	(57.8	(86.6
1	70	5%)	9%)	%)	5%)	4%)	5%)	3%)	6%)	3%)	2%)
B	42	3976	3885	908	1953	1932	1941	1944	1457	2427	3696
4	72	5403	6631	772(	1769	4862	3440	3191	7755	8876	5692
_	43	(93.0	(90.9	2.13	(45.7	(45.2	(45.4	(45.5	(34.1	(56.8	(86.5
2	40	7%)	5%)	%)	2%)	3%)	4%)	1%)	2%)	3%)	2%)
B	45	4237	4131	106	2072	2059	2063	2068	1546	2585	3945
4	43	9801	9748	005	1563	8185	9398	0350	4325	5423	7380

_3	6794	(93.27%)	(90.94%)	3(2.33%)	(45.61%)	(45.33%)	(45.42%)	(45.51%)	(34.03%)	(56.9%)	(86.84%)
BN_1	42851398	39812862(92.91%)	38985037(90.98%)	827825(1.93%)	19552779(45.63%)	19432258(45.35%)	19485675(45.47%)	19499362(45.5%)	13968007(32.6%)	25017030(58.38%)	37161642(86.72%)
BN_2	46317896	43130598(93.12%)	42228124(91.17%)	902474(1.95%)	21166103(45.7%)	21062021(45.47%)	21100957(45.56%)	21127167(45.61%)	14742993(31.83%)	27485131(59.34%)	40427294(87.28%)
BN_3	46268788	42981710(92.9%)	42104916(91.0%)	876794(1.9%)	21085639(45.57%)	21019277(45.43%)	21041338(45.48%)	21063578(45.52%)	14908292(32.22%)	27196624(58.78%)	40365798(87.24%)

Notice: sample: sample name; total_reads: the number of clean reads of sequencing data after quality control; total_map: the number and percentage of reads compared to the genome; unique_map: The number and percentage of reads compared to the unique position of the reference genome (used for subsequent quantitative data analysis reads); multi_map: The number and percentage of reads compared to multiple positions in the reference genome read1_map: the number of read1 compared to the reference genome and its percentage read2_map: the number of read2 compared to the reference genome and its percentage; positive_map: the number and percentage of reads aligned to the positive strand of the reference genome; negative_map: the number and percentage of reads compared to the negative strand of the reference genome; splice_map: the number and percentage of reads split and aligned to the genome; unsplice_map: The number and percentage of reads that have not been split and aligned to the genome; proper_map: the number of reads and their percentages of the paired read1 and read2 simultaneously aligned to the genome.

Table S3. The first three differential genes up-regulated.

gene_id	A0_1_count	A0_2_count	A0_3_count	A2_1_count	A2_2_count	A2_3_count	A4_1_count	A4_2_count	A4_3_count	AN_1_count	AN_2_count	AN_3_count	B0_1_count	B0_2_count	B0_3_count	B2_1_count	B2_2_count	B2_3_count	B4_1_count	B4_2_count	B4_3_count	BN_1_count	BN_2_count	BN_3_count
MD03G1231800	6418	4764	6144	7574	5217	6306	5939	5576	5243	1817	2351	2239	3453	4312	3985	14345	9388	7435	13354	13404	14779	1906	1360	1768
MD09G1	49	1453	35	8853	2150	6822	14	42	112	168	1183	14	1195	1319	1210	30778	7012	7589	29112	14706	17015	858	878	827

075000																								
MD11G10095300	1291	5262	1049	9676	5179	6339	1084	2943	1601	3573	4109	1537	2101	664	568	12883	7329	2220	9764	15698	17452	1351	1391	1523

TableS4.The first three differential genes up-regulated.

Gene_ID	A0_1_cou	A0_2_cou	A0_3_cou	A2_1_cou	A2_2_cou	A2_3_cou	A4_1_cou	A4_2_cou	A4_3_cou	AN_1_cou	AN_2_cou	AN_3_cou	B0_1_cou	B0_2_cou	B0_3_cou	B2_1_cou	B2_2_cou	B2_3_cou	B4_1_cou	B4_2_cou	B4_3_cou	BN_1_cou	BN_2_cou	BN_3_cou

	nt	nt	nt	nt	nt	nt	nt	nt	nt	nt	nt	nt	nt	nt	nt	nt	nt	nt	nt	nt	nt	nt	nt	nt
MD16G1102700	97323	48942	99424	28120	79207	58762	91427	104649	96242	136058	116437	167732	167070	206564	197255	26788	98134	108188	5513	11572	11881	168417	150294	195189
MD13G1134600	35297	44837	26143	24021	36143	35864	27257	40953	41732	31913	26055	33204	126913	168401	145498	31762	99802	90392	16921	19361	18730	112542	127326	122955
MD0	716	144	541	198	956	259	101	955	872	954	886	756	179	198	186	260	791	106	624	973	117	132	155	144

4	1	6	5	8	1	1	0	0	8	1	1	5	4	2	8	5
G					3	5	3	6	1	2			7	4	2	3
1					0	2	2			3				7	2	2
0																
6																
4																
2																
0																
0																

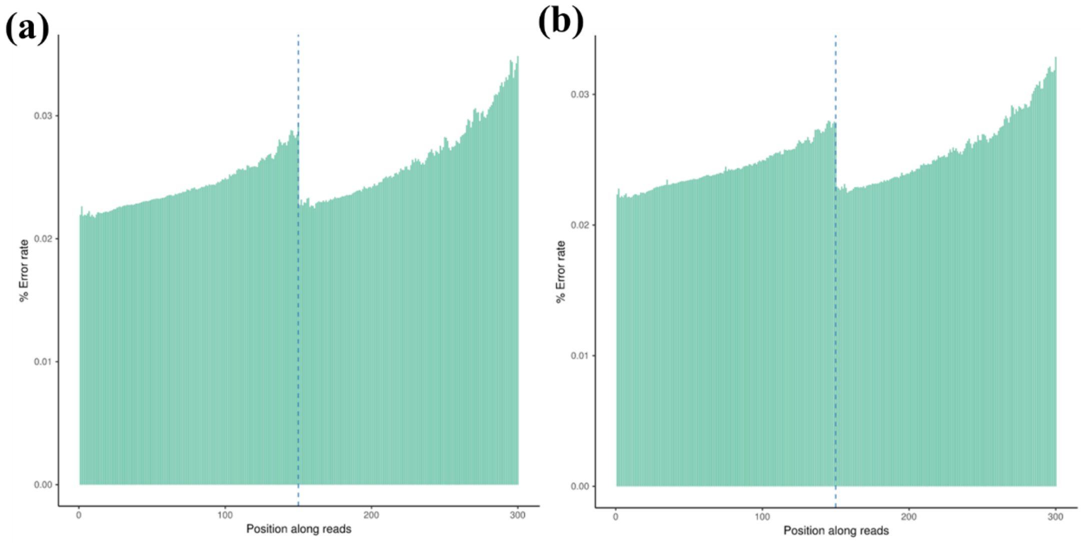

Figure S1. Sequencing error rate distribution. a, AN; b, BN. In the figure, the abscissa is the base position of reads, and the ordinate is the single-base error rate.

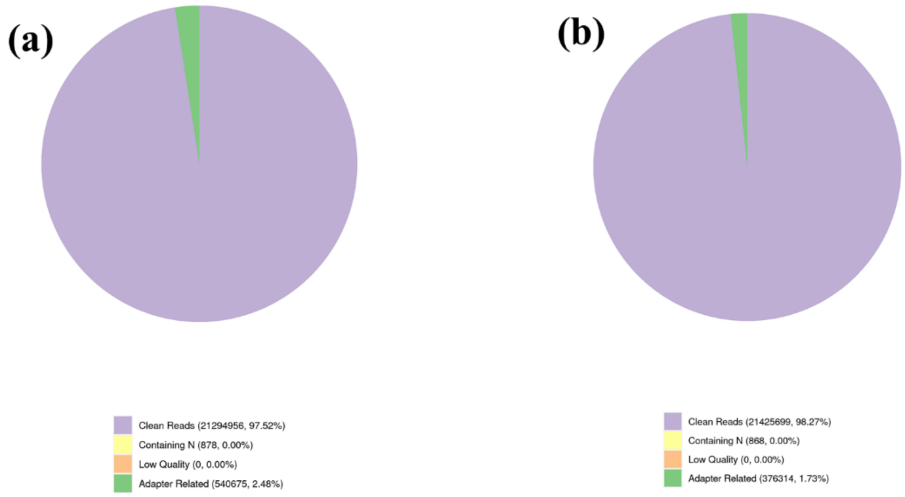

Figure S2. Sequencing data filtering results. a, AN; b, BN.

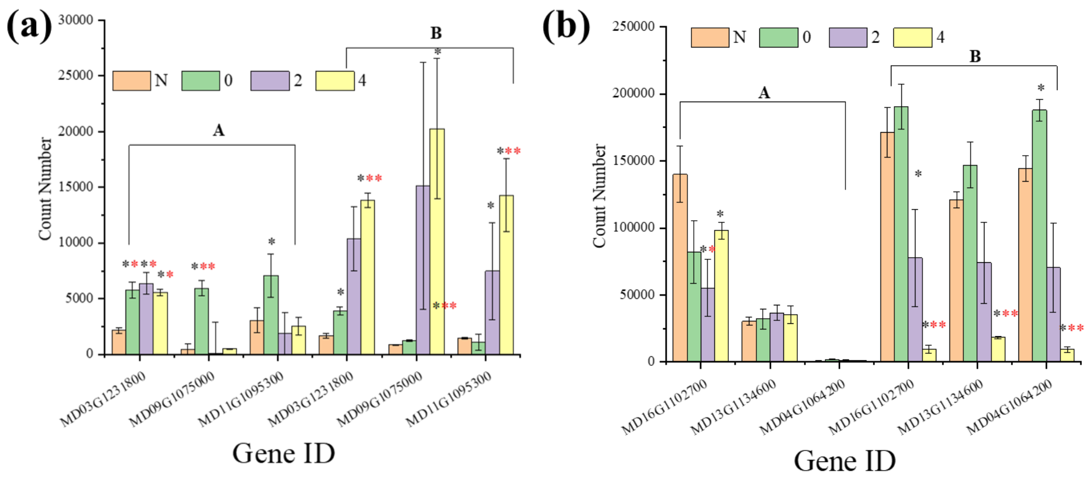

Figure S3. The count number statistics of the first three differential genes. a, up-regulated gene statistics; b, down-regulated gene statistics.Statistical difference: *, **, *** represent the results with p values less than 0.05, 0.005 and 0.001, respectively.

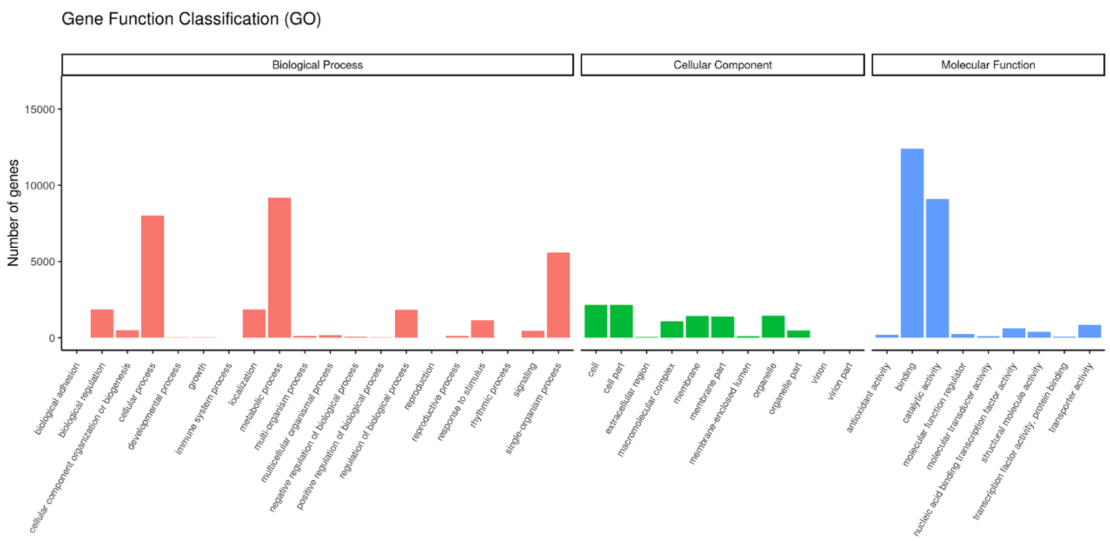

Figure S4. GO enrichment analysis of the overall sample.
